# SMILES alignment: a dynamic programming approach for the alignment of metabolites and other small organic molecules

**DOI:** 10.1186/s12859-025-06278-y

**Published:** 2025-10-17

**Authors:** Alexis L. Tang, David A. Liberles

**Affiliations:** Center for Computational Genetics and Genomics and Department of Biology, Temple University, 1900 N. 12Th Street, Philadelphia, PA 19122 USA

**Keywords:** Dynamic programming, Chemical alignment, Global alignment, Organic molecule similarity search, Biochemical pathways, Chemoinformatics, Evolutionary biochemistry

## Abstract

**Background:**

There is a need for computational approaches to compare small organic molecules based on chemical similarity or for evaluating biochemical transformations. No tool currently exists to generate global molecular alignments for small organic molecules. The study introduces a new approach to molecular alignment in the Simplified Molecular Input Line Entry System (SMILES) format. This method leverages programming and scoring alignments to minimize differences in electronegativity, here using a measure of atomic partial charges to address the challenge of understanding structural transformations in reaction pathways. This can be applied to study transitions from linear to cyclical pathways.

**Results:**

The proposed method is based on the Needleman-Wunsch algorithm for sequence alignment, but it uses a modified scoring function for different input data. Validation against a benchmarked dataset from the Krebs cycle, based on the known chemical transformations in the pathway, confirmed the efficacy of the approach in aligning atoms that are known to be the same across the transformation. The algorithm also quantified each transformation of metabolites in the Pentose Phosphate Pathway and in Glycolysis. The method was used to study the difference in chemical similarity over transformations between linear and cyclical pathways. The study found a midpoint dissimilarity peak in cyclical pathways (particularly the Krebs Cycle) and a progressive decrease in molecular similarity in linear pathways, consistent with expectations.

**Conclusions:**

The study introduces an algorithm that quantifies molecular transformations in metabolic pathways. The algorithm effectively highlights structural changes and was applied to a hypothesis about the transition from linear to cyclical structures. The software, which provides valuable insights into molecular transformations, is available at: https://github.com/24atang/SMILES-Alignment.git

## Introduction

Sequence alignment for biological macromolecules like DNA and proteins is a common precursor to the analysis of these molecules [1]. Sequence analysis is based upon the notion of identifying homology or descent from a common ancestor but is predicated on the signal for this lying in chemical similarity which is maximized with position-specificity to find an optimal alignment [1]. Except for biochemical transformations, the notion of homology is absent in the alignment of small organic molecules, but the tools for sequence alignment can be reharnessed to align small organic molecules based upon atomic similarity, with position-specific detail. Applications of molecular alignment can include basic questions in biochemistry, including in evolutionary biochemistry, as well as any number of applications in fields from organic synthesis to pharmacology.

Chemical similarity algorithms and programs form the core of various applications such as drug discovery, chemical database management, and structural prediction [1]. Tools like RDKit [2] and Open Babel [3] have provided robust frameworks for generating molecular fingerprints, a popular method for comparing chemical structures. These fingerprints, bit vectors representing the presence or absence of specific structural features, can be compared using metrics such as Tanimoto or Dice coefficients [4], which are simple proportions. Graph-based methods, which treat molecules as graphs with atoms as nodes and bonds as edges, are another common strategy [5]. Algorithms such as the Maximum Common Subgraph (MCS) find the largest shared structure between two molecules [6]. These are approaches for comparing the presence or absence of atoms and bonds. In recent years, machine learning methods have emerged as powerful tools for predicting chemical similarity. Neural networks trained to predict molecular properties directly from molecular graphs or SMILES strings have shown promising results [7, 8]. Moreover, Hadipour et al. introduced a framework for clustering many small molecules by generating embeddings of global chemical properties and local atom and bond features using variational autoencoder-based and principal component analysis-based methods [9]. Using K-means clustering on these embeddings, the framework grouped over 47,000 molecules into biologically relevant clusters, highlighting the potential of neural networks in chemoinformatics applications [9].

Molecular comparisons can consider both shared atom and shared bond information, with some methods focusing on atomic comparisons including neighbors. Conceptually from a chemical perspective, molecular comparisons would benefit from bond-centric approaches that include the atoms being bonded and the type of bond rather than simpler atom centric approaches.

Despite these advances, most existing methods do not explicitly incorporate key physicochemical properties like partial charges, as determined by electronegativity, into their similarity measures. Moreover, handling gaps in alignment remains a challenging issue, but it is necessary for the generation of a global alignment comparing molecules and is specific to the method presented here. The more common metric used in bioinformatics and chemoinformatics to calculate the similarity of molecular structures is the Tanimoto coefficient, which is a simple presence/absence proportion [10] and does not offer all the features of a full alignment, particularly for cases where biochemical transformations enable a definition of site homology, such as cases where the identical atom has been established using isotope labeling.

The Tanimoto Coefficient is simple and can be used to traverse through large databases, however this simplicity is the source of many criticisms. One significant concern is its scale dependency, as it can produce differing results based on the size of the molecules being compared, making it a less ideal choice for contrasting larger molecules with smaller ones [11]. The similarity for smaller structures might be overly amplified [11], although one can conceptually normalize by the number of atoms considered. Furthermore, the coefficient’s binary nature means it doesn’t account for the actual count of a specific feature within a molecule; it considers a feature that appears once and ten times in two different molecules as identical [11]. Despite giving a quantitative measure of similarity, it doesn’t offer a direct interpretation concerning specific chemical attributes, bonds, or structures contributing to the similarity. Also, there is no well-defined rule for setting a threshold for similarity or dissimilarity, with different studies using different considerations [12]. The definition of “similar” can vary according to different contexts [12]. Whereas a Tanimoto Coefficient does not allow calculation of a true molecular distance for these reasons, methods based upon an explicit alignment enable calculation of a precise distance between molecules after transformations. Further, duplication of fingerprints would not be quantified in a Tanimoto Coefficient, even though these can be an important part of a chemical transformation. Because of this, alignment-based methods have capabilities that are not represented in the simpler summary measures.

Clustering methods for chemical similarity, such as CAST, Jarvis-Patrick, and Raymond-Willett, are available for various applications [13–15]. These methods include adjustable parameters that allow fine-tuning for specific datasets and clustering requirements, handling diverse data types like gene expression patterns and chemical structures [13–15]. They also manage overlapping clusters, as seen in the fuzzy clustering techniques like the Raymond-Willett method, which is advantageous when data points belong to multiple groups [15]. Additionally, hierarchical methods like Ward’s Algorithm use objective validation indices, such as Kelley’s Index, to select the best clustering [16]. However, these methods face challenges, particularly in computational complexity, making them resource-intensive for large datasets and reducing their efficiency in high-throughput or large-scale applications [15]. Their performance is highly sensitive to parameter choices, requiring careful tuning and potentially extensive trial and error [15]. Scalability issues arise as some methods may not perform well with very large datasets or high-dimensional data, limiting their applicability in big data contexts [15]. The complexity in setting and interpreting multiple adjustable thresholds, especially in methods like Raymond-Willett, can complicate the clustering process [15]. Techniques employing greedy algorithms, such as Raymond-Willett, may not always find the global optimum, potentially leading to suboptimal outcomes [15].

The MOS (Maximum Overlapping Set)-based compound clustering approach improves upon traditional MCS (Maximum Common Substructure) techniques by calculating the largest set of substructures within a dataset [17]. This method makes fine-grained distinctions between atom types and incorporates empirical correction terms [17] These enhancements significantly improve the method’s performance in grouping compounds according to their biological targets, particularly in HTS (High throughput Screening) scenarios [17]. The approach works well in identifying biologically meaningful relationships between chemical structures from different classes, essential for uncovering subtle similarities missed by classical substructure-based analyses [17]. Additionally, it increases confidence in single hits by finding structural relationships with other compounds [17]. Despite its advancements, the MOS-based method has drawbacks. It struggles with scalability, efficiently handling up to 3,000 structures but becoming cumbersome with datasets around 10,000 structures or more [17]. The computational complexity of the clique detection algorithms also needs improvement for faster processing on standard computing clusters [15, 17]. The method doesn’t account for the statistical relevance of random similarities, as common chemical features can lead to misleading similarities [17]. Empirical correction terms, while beneficial, do not capture all topological information [17]. There is also a need for a penalty term to address the occurrence of many small fragments in the calculated MOS, which can lead to less meaningful clustering results [17]. Lastly, this method does not generate a global molecular alignment.

More recently, new methodologies such as iterative Random subspace PCA clustering (iRaPCA) and Silhouette Optimized Molecular Clustering (SOMoC) have demonstrated high performance in clustering small molecules across many datasets, outperforming well-known clustering methods such as Ward and Butina in terms of both within-cluster and between-cluster distances [18]. iRaPCA is an iterative approach based on K-means optimization, Principal Component Analysis, and a random subspace approach [18]. SOMoC draws on molecular fingerprinting, dimensionality reduction via the Uniform Manifold Approximation and Projection (UMAP), and clustering with the Gaussian Mixture Model (GMM) [18]. Despite these advancements, there are limitations to both methods. They rely on hyperparameters that can be finetuned to reach an optimal clustering, potentially causing extensive trial and error [18]. Moreover, the GMM algorithm used in the SOMoC method requires a minimum sample size to accurately estimate the parameters of the distributions. Spectral clustering has also emerged as a promising alternative. Molecules are divided into groups based on the eigenvectors of a similarity or affinity matrix, which can be used to capture complex molecular relationships in chemical space [19]. This method has been parameterized for various molecular descriptors and datasets [19].

The Atom–Atom-Path (AAP) similarity metric prioritizes fragment hits by rewarding common substructures and recognizing minimal structural differences between molecules [20]. This method encodes each atom with linear paths extending up to seven bonds and computes the similarity between atoms by comparing these paths. The algorithm then maps each atom in the smaller molecule to a unique atom in the larger molecule, maximizing the sum of atom-to-atom similarities. This results in a similarity score that reflects the local alignment of molecular substructures [20]. AAP similarity is particularly sensitive to small changes in substitution patterns, enabling it to generate fragment-sized molecules [20]. However, it is computationally intensive compared to simpler methods, posing scalability issues for large datasets. It also doesn’t incorporate all topological information [20].

Evaluating molecular similarity is an important step towards the goal of generating a global alignment of molecules. While other algorithms for evaluating molecular similarity focus on structural similarities and empirical corrections, when they represent atoms via properties such as their atomic numbers, they fail to capture their chemical similarity. On the other hand, partial charges provide a measurement that reflects how chemically similar two atoms are in the context of a molecule. Substituting an atom with another of similar partial charge should incur a lower cost than substituting one that differs more, akin to amino acid changes in evolutionary biology with different propensities. These methods could be used to identify core regions to anchor an alignment, analogous to Partial Order Alignment (POA) for sequences [21]. The more straight forward approach is to start with basic foundational techniques like the Needleman-Wunsch algorithm, which is ultimately needed in approaches like POA to align between anchor points. This approach is known to generate global alignment optima, which is the core problem addressed here.

Here, we use a partial charge measure as a core feature of atomic behavior within molecules towards a scoring function to align molecules, establishing correspondences between atoms or groups. By integrating Gasteiger charges into the scoring function, it offers a more detailed perspective on atomic interactions and relationships, making it a valuable addition to the toolkit for understanding and analyzing molecular structures and transformations.

Gasteiger charges, also known as Gasteiger-Marsili charges, are partial atomic charges calculated through an empirical method [22]. These charges approximate the partial charges that would be obtained from a full quantum mechanical treatment of a molecule [22]. The calculation method is based on the electronegativity equalization method (EEM) [22]. The Gasteiger-Marsili partial charge computation returns a set of partial charges for each atom in the molecule, resulting in an output of a list (or other data structure) of these partial charges, one for each atom in the molecule [22].

The Gasteiger charge computation involves an iterative procedure where charges are redistributed among connected atoms in each step, considering their different electronegativity and hardness values [22]. The process of calculating Gasteiger charges begins with an initial estimation of the partial atomic charges based on the atom type.This estimation is typically derived from a lookup table that contains precalculated charges for different atom types [23]. This initial estimation is based on the principle of electronegativity equalization, an empirical observation that, in a stable molecule, the electronegativity of each atom tends to equalize with that of the surrounding atoms [22]. The method then employs an iterative procedure to adjust the initial charges until the total potential of the molecule is minimized [22]. An iterative method adjusts atomic charges based on electronegativity differences between bonded atoms [22]. For each bond, the charge differences are computed and proportionally applied to the atoms involved, maintaining the molecule’s overall neutrality [22]. This iterative adjustment continues until minimal charge changes are observed, indicating convergence [22]. Subsequently, the calculated charges are normalized to match the molecule’s total charge [22]. Readily available Python modules exist to calculate such charges, such as RDKit [2].

The Needleman-Wunsch algorithm is a dynamic programming technique for globally aligning two protein or nucleotide sequences. It optimizes alignment by maximizing a scoring system, which rewards matches and penalizes gaps or mismatches between the sequences (based upon expectations of change, insertion, or deletion) [24]. Gap penalties are divided into two different categories: a gap opening penalty when introducing a new gap and a gap extension penalty when lengthening an existing gap [24]. The algorithm uses three matrices (M, X, Y) to score aligned pairs, gaps in the first sequence, and gaps in the second sequence, respectively, accommodating both gap introduction and extension [24].

$$ \begin{aligned} M(i,j) = & \,\max \{ M(i - 1,j - 1),X(i - 1,j - 1),Y(i - 1,j - 1)\} + s(a_{i} ,b_{j} ) \\ X(i,j) = & \,\max \{ M(i - 1,j) - g_{{{\text{open}}}} ,X(i - 1,j) - g_{{{\text{extend}}}} \} \\ Y(i,j) = & \,\max \{ M(i,j - 1) - g_{{{\text{open}}}} ,Y(i,j - 1) - g_{{{\text{extend}}}} \} \\ F(i,j) = & \,\max \{ M(i,j),X(i,j),Y(i,j)\} \\ \end{aligned} $$

During the matrix filling phase, starting from the initial positions (M(1,1), X(1,1), Y(1,1)), the algorithm fills each matrix based on recursive relations, moving through the sequences until it reaches the end (M(n,m), X(n,m), Y(n,m)), with n and m representing the lengths of the sequences [24]. This process calculates optimal scores for aligning characters or introducing gaps, ensuring each cell captures the best score up to that point [24].

The traceback method then identifies the optimal alignment starting from the bottom-right corner (F(n,m)), where F represents the highest score among M, X, and Y for each cell [24]. Tracing back according to the source of each cell’s score, the algorithm reconstructs the alignment, moving diagonally for matched characters or vertically/horizontally for gaps, until it reaches the start, yielding the final optimal alignment [24].

Our approach is centered around the creation of scoring matrices that are based on Gasteiger charges, drawing inspiration from the BLOSUM matrices utilized in protein sequence alignment by taking log-odds scores for transformation based upon chemical similarity (in the case of BLOSUM mediated by an evolutionary process) and can be used in the same way as part of the alignment procedure [25]. We constructed two distinct types of scoring matrices: the first compares all the partial charges in the metabolism database, while the second focuses on the charges of specific atom pairs such as (C,O), (C,C), and (O,O) within this database [26]. Building upon this groundwork, we applied the Needleman-Wunsch algorithm, a standard tool in bioinformatics for sequence alignment, to enable the alignment of SMILES molecules using our specially developed scoring matrices [24]. To validate our algorithm, we applied it to a known cyclical biochemical pathway, the Krebs cycle, as a truth set. This application not only tests the algorithm’s precision and reliability but also establishes a benchmark for its usage in analyzing more complex molecular pathways. Finally, we employed the algorithm to examine the Pentose Phosphate Pathway. This step demonstrated the practical applications of our method in biochemical and molecular research, providing new insights into molecular interactions and alignment within this pathway.

## Methods

In this study, focusing on metabolic pathways is of crucial importance as these sequences of reactions form the central framework of biochemistry, offering a rich context to align molecules based on their role in shared or similar chemical reactions. Understanding the similarities and differences between molecules in these pathways can offer valuable insights into metabolic processes and their evolution, facilitating an in-depth analysis of the dynamic nature of metabolic reactions. The alignment of molecules from metabolic pathways can potentially help decipher the molecular basis of transitions between linear and cyclic modes under varying cellular conditions, a critical aspect in predicting metabolic behavior.

### Database

The study analyzed molecules from metabolic pathways using SMILES strings focusing on the Metabolism Pathway from Reactome (R-HSA-1430728) containing 1101 molecules [26]. To ensure consistency and accuracy in representing these metabolic molecules, each molecule was represented by its SMILES string. The SMILES strings recovered from PubChem were canonized using Indigo python package, ensuring a standardized description of the molecular structures [27].

### Canonization

Canonization of SMILES representations is essential for the successful application of alignment algorithms in the comparison of metabolic pathways. In the absence of canonical forms, different representations of the same molecule may yield inconsistent and unreliable alignment scores, complicating the interpretation of results. Canonical SMILES ensure a consistent string representation for the same molecular structure, irrespective of the way it was initially encoded. Blind importation of pathways may be insufficient, proper data processing is necessary.

The stability of canonical SMILES strings when subjected to small, random changes was investigated using the canonization script (found in the GitHub repository). This aims to understand how minor alterations affect the canonical SMILES representation and evaluate the consistency of the canonicalization process across similar molecules. Ensuring that canonical SMILES provide a unique and consistent representation of molecules is crucial for maintaining reliability in the molecular alignment.

This process begins by defining a list of parent molecules (the molecules in the Krebs cycle was used) represented as SMILES strings. For each parent molecule, variant molecules are generated by systematically replacing an oxygen atom with a sulfur atom. Both the parent molecules and their variant molecules are then canonicalized to ensure a standardized comparison, but only after the variant has been made. The Levenshtein distance, a measure of string similarity, and Tanimoto distance were calculated between each parent molecule and its variants, quantifying the changes in the canonical SMILES due to the introduced variations. The average Levenshtein and Tanimoto distance between each parent molecule and its variants is then computed.

AllChem Module and Gasteiger charges:

There are two scoring matrices that were created for the final alignment: an All vs All and Paired scoring. All_vs_All_Scoring.py procures SMILES representations of molecular structures of each molecule in the metabolism database, from which it computes the Gasteiger charges, quantities that encapsulate the relative electronegativities of atoms. The obtained charges are aggregated into a master list containing all charges in all molecules. Then, non-finite values (NaN and infinities) are filtered out and the corresponding atoms are removed from the analysis. In the Gasteiger charge calculations using the AllChem module, encountering NaN (Not a Number) or infinite results usually signifies some anomaly or error during the computational process. There are several potential reasons for this. If the molecular structure inputted is invalid or misrepresented, the calculation could fail, resulting in a NaN or infinite value. Similarly, the Gasteiger method relies on predefined assumptions about the atomic constitution and bonding of molecules. If the input molecule encompasses unusual atom types or atypical bonding scenarios, these outliers might cause an error in charge calculation, returning NaN or infinite. Further, numerical computation issues like division by zero or overflow/underflow complications during the iterative process of the Gasteiger method could also yield these aberrant results.

**Fig. 1:**
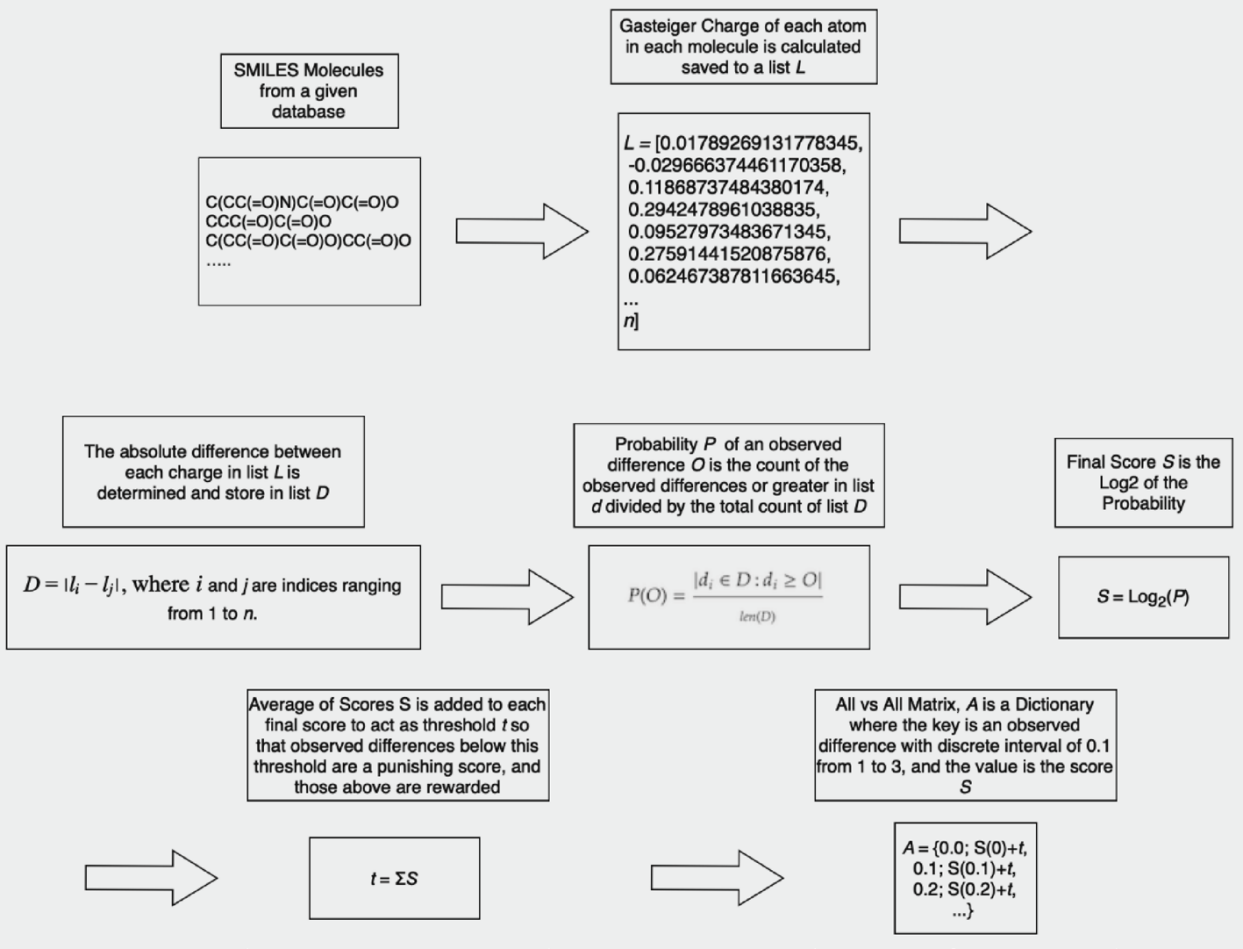
Calculation method of All vs All scoring matrix: The figure shows the calculations of a scoring matrix that quantifies the likelihood of charge differences between pairs of atoms. Charge differences are calculated and binned into intervals of 0.1 from 0 to 3, with the probability for each interval determined by the base-2 logarithm of their relative frequency. The matrix promotes minor electronegativity variations with a rewarding score and penalizes larger discrepancies

The absolute difference between every possible pair of charges is determined, generating an exhaustive list of charge differences. A probability is determined by dividing the count of charge differences greater than or equal to a given interval by the total number of observations. The score for each interval is then determined by taking the base-2 logarithm of this probability. To achieve a balance between computational efficiency and accuracy, the range of observable charge differences is discretized into intervals of 0.1, spanning from 0 to 3. For each of these discrete intervals, the relative frequency of observed charge differences within that interval is computed. As a result, a scoring matrix is constructed, which provides a quantitative measure of the likelihood of any two atoms having a certain charge difference. The primary objective of the scoring matrix is to incentivize minor partial charge variations while penalizing significant discrepancies between aligned atoms. To establish a clear benchmark for this, the mean of all computed differences was ascertained. This average serves as the threshold in the scoring matrix; differences exceeding this average are met with penalties, while those below it are awarded favorable scores. The calculation is summarized in Fig. 1.

Paired_Scoring.py is dedicated to the generation of a paired scoring matrix. The scoring function within this structure is based on the differences of Gasteiger charges between paired atoms. The objective is to uncover any underlying trends or patterns exclusive to these specific atomic pairs, such as all Carbon–Carbon (C–C), Carbon–Oxygen (C-O), etc., across all molecules in the database. By comparing these charge differences across a wide range of molecules, the study aims to identify potential trends or phenomena that might enhance our understanding of the behavior of these atoms within the realm of metabolic reactions that the All vs. All scoring matrix is not capable of. For each atom pair, a scoring matrix similar to that in All_vs_All_Scoring.py is created, with the difference that it bases its scores on the specific pair’s charge differences.

Other methods of charge calculation can be seamlessly integrated into the algorithm if an alternative method for charge calculation is preferred.

### Algorithm

All_vs_All_Alignment.py focuses on the sequence alignment of two molecules represented in the SMILES format by use of the Needleman Wunsch alignment algorithm [24]. This script enables the execution of the standard Needleman-Wunsch Algorithm with unequal gap initiation and extension penalties. Before aligning the molecules, the script processes the SMILES by stripping the characters and then aligns based on the partial charge pattern of the molecule. Once the alignment is achieved, the original characters are replaced. This is done so that non-characterizable elements (bonds, charges, and rings) do not interfere with the alignment. The align() function in All_vs_All_Alignment.py is the Needleman-Wunsch alignment algorithm. When the scoring matrices are being filled, the scores are determined by the difference of charge of the two atoms being aligned using the designed scoring matrix. This is distinct from the traditional manner where the score between two amino acids or base pairs is determined by match or mismatch scores. Align() returns the two aligned molecules and an alignment score. Paired_Alignment.py carries a notable distinction from All_vs_All_Alignment.py as it utilizes the scoring matrix devised in Paired_Scoring.py.

### Pathways

The Krebs cycle, also known as the citric acid cycle or tricarboxylic acid cycle, was chosen for the validation of the algorithm due to several reasons. Primarily, it is one of the most well-studied and understood biochemical pathways in cellular metabolism. This high level of understanding ensures that the true transformations and mechanisms involved in each step are well-documented, providing a solid ground truth against which to compare the algorithm’s predictions. Furthermore, the Krebs cycle is relatively short with eight distinct enzymatic steps, simplifying the process of validation [28]. Each step in the cycle involves the transformation of one molecule into another, which aligns well with the purpose of the algorithm: to compare and align molecular structures [28]. In this case, similarity is not the only information but is also a proxy for the underlying signal of site homology. Moreover, the cyclical nature of the Krebs cycle provides additional benefits for validation. In a cycle, the product of the final step is the substrate for the first step [28]. This allows for a unique validation perspective: not only can individual transformation accuracy be identified but also the ability of the algorithm to correctly align and track transformations (which carbon is the same through enzymatic steps) across the entire cycle. Overall, the combination of the Krebs cycle’s simplicity, cyclical nature, and well-established scientific understanding makes it an ideal choice for validating the accuracy and utility of the molecular alignment algorithm. It can also be used to generate data to address evolutionary questions about the evolution of new enzymatic steps and the degree of chemical transformation that typically happens in a step, including questions of if and how linear pathways can become circular ones or about the retrograde hypothesis for pathway evolution [29].

In the approach, coenzymes were excluded from the analysis. This decision was driven by the role coenzymes typically play in the metabolic process. Often acting as electron or functional group carriers, coenzymes do not structurally transform in the same way primary metabolites do [28]. Their inclusion could introduce unnecessary complexity, potentially obscuring the transformation patterns being studied.

To ensure consistency and maintain accurate representation of the Krebs cycle environment in our truth dataset, we standardized the molecular representations using the Indigo toolkit [27]. This process consistently encodes all molecules with specific functional groups, such as carboxylate groups, in their SMILES string representations. As a result, the final dataset featured uniform SMILES strings where the order of atoms, functional groups, and carbon chains were uniformly presented, facilitating clear discernibility and reliable computational analysis.

### Validation

**Fig. 2:**
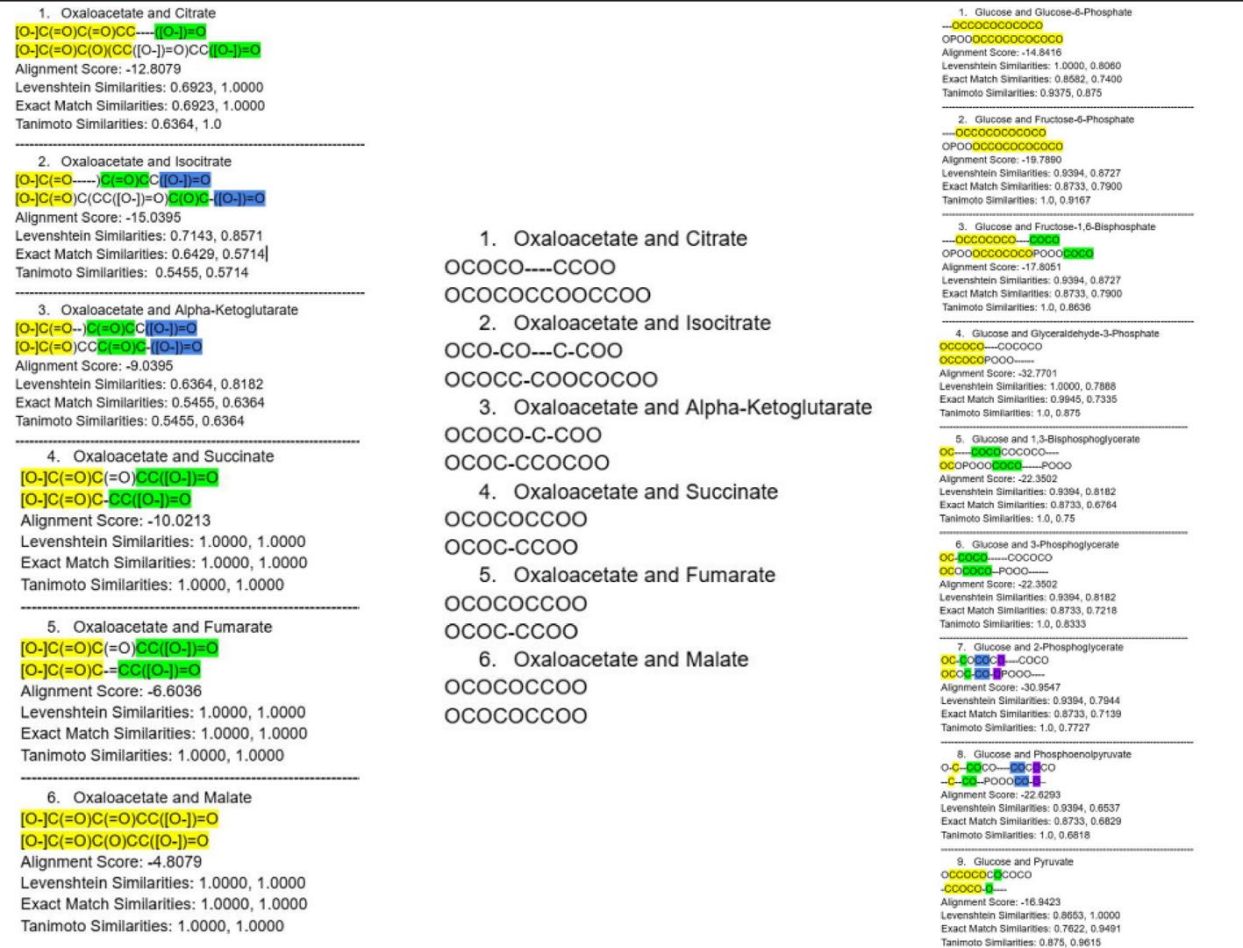
Alignment of Oxaloacetate in the Krebs Cycle using All_vs_All_Alignment (Left). Sample of the Truth Dataset of the Krebs cycle without SMILES characters (Middle). Left figure presents the alignment of oxaloacetate using All_vs_All_Alignment and the middle figure demonstrates what the algorithm will interpret. Right figure is the alignment of glucose using the all_vs_all in glycolysis

The validation of the alignment algorithms was conducted using a carefully curated truth dataset centered on the Krebs cycle. The Krebs cycle, integral to the metabolic functions of aerobic organisms, was streamlined by removing coenzymes and focusing exclusively on the seven primary metabolites [28]. Each metabolite was aligned with the other metabolites. The purpose of aligning all molecules with each other is to validate the algorithms’ ability to differentiate changes each molecule makes as it progresses through the metabolic cycle. An exhaustive analysis of the seven metabolites’ structure and composition was undertaken, with crucial carbons and functional groups identified [23, 28]. Figure 2 shows an example of the validated alignment process of the first metabolite, Oxaloacetate, aligned with the other metabolites, where the left shows the entire SMILES aligned and the middle shows what the algorithm will interpret. It is worth noting that for each glucose molecule metabolized, the citric acid cycle completes two revolutions [28]. Nevertheless, for the purpose of simplicity and to maintain a clear focus on the fundamental transformations, the validated alignment was restricted to the first cycle only.

A second validation using the same methodology was conducted using glycolysis, a linear metabolic pathway crucial for cellular respiration [28]. Glycolysis is the process by which glucose, a six-carbon sugar molecule, is broken down into two three-carbon molecules of pyruvate. This pathway consists of ten enzyme-catalyzed reactions that occur in the cytoplasm of the cell, making it a fundamental aspect of both aerobic and anaerobic respiration [28]. An example of glucose (the first molecule) being aligned with the rest of glycolysis is seen in the right of Fig. 2.

While the input of the align function is the SMILES with the characters, the alignment itself is done based on the partial charge blueprints (a list of partial charges determined from a SMILES molecule with non-atomic characters that were removed) where the non-atomic characters are stripped. The alignment itself will be compared to those on the right set of alignments in Fig. 2.

The validation of the optimal parameters involved a comprehensive examination of the alignment parameters — gap open and gap extension penalties. The range for these parameters was chosen between − 5 and + 5 to ensure a broad coverage of possible scenarios and enhance the robustness of the optimal parameters. Every possible combination of these parameters was tested, and the results are generated in a CSV file when either alignment program is run. This approach was designed to identify the most fitting set of parameters that yielded the highest alignment scores (higher scores indicating more similarity). Based on existing knowledge and the fundamental principles of sequence alignment, certain expectations were put forward [24]. The gap open penalty was anticipated to be more penalizing than the gap extension penalty for similar alignments. This is based on the understanding that introducing a gap in the alignment is a more significant event and should be penalized more heavily than simply extending an already opened gap [24]. The comprehensive testing allowed for a rigorous examination of these expectations and served as a robust strategy for the identification of the most fitting parameters for this particular alignment problem.

The optimal parameters were determined by using two methods of similarity (Levenshtein similarity and exact similarity) when comparing the validated alignments determined previously with the experimental alignments from the Krebs Cycle Levenshtein similarity and exact similarity, [25, 30]. The parameters identified as optimal were those that accurately aligned the metabolites in the Krebs cycle compared with known biochemical transformations.

## Results

### Canonization

**Fig. 3:**
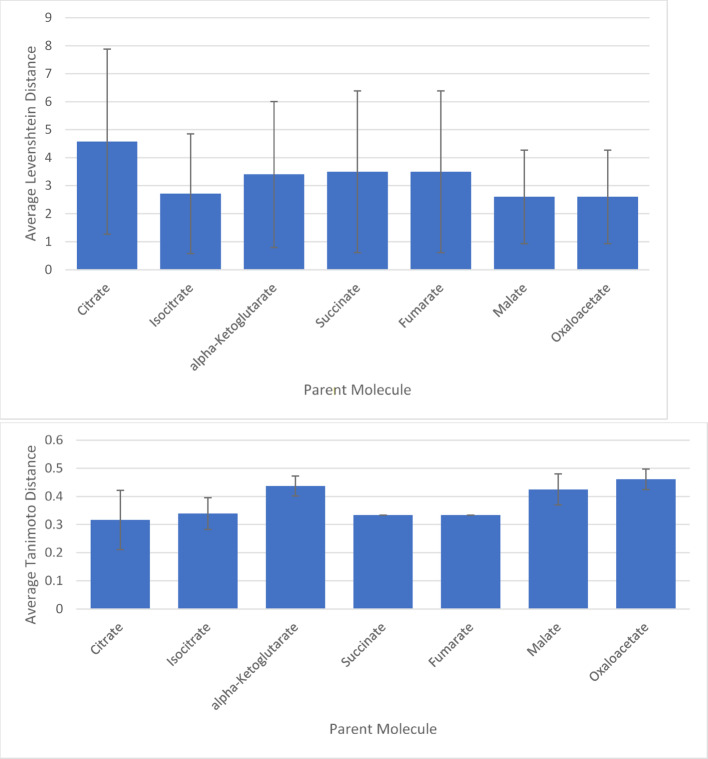
Average Levenshtein (Top) and Tanimoto (Bottom) Distance Between Parent Molecules and Their Variants. The figureplots the average Levenshtein and Tanimoto distances for parent molecules and their variants, thereby illustrating the stability of canonical SMILES strings under small, chemically valid variations. The error bars represent the standard deviation of the Levenshtein and Tanimoto distances between each parent molecule and its generated variants, providing a measure of the variability in the distances. Standard Deviations for Levenshtein: [3.3, 2.1, 2.6, 2.9, 2.9, 1.7, 1.7]. Standard Deviations for Tanimoto: [0.1, 0.06, 0.04, 0, 0, 0.06, 0.04]

The alignment method relies upon canonization generating the same atomic order for the same and similar molecules. This assumption was tested. Figure 3 indicates that systematically changing an oxygen to a sulfur in a set of metabolites has the potential to impact the reliability of the canonization process. While canonization was controlled in cases where the biochemistry is known in this study, users need to evaluate the canonization of molecules in their analysis.

### All vs all scoring and alignment

**Fig. 4:**
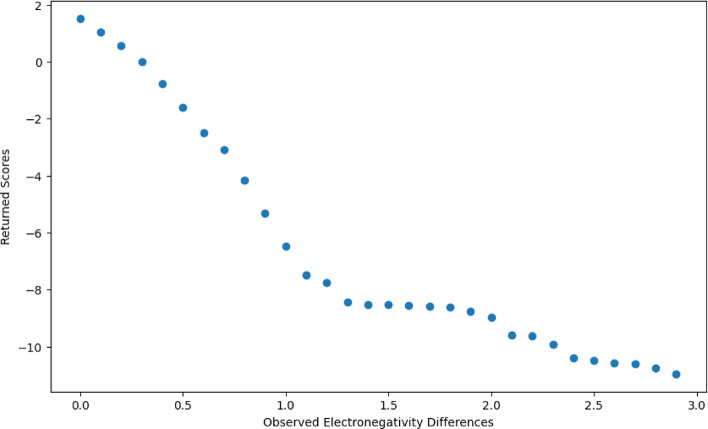
All vs. All scoring Matrix Plot. Log2 probability of observed Gasteiger charge differences or greater in 0.1 increments

Figure 4 shows the plotted All vs. All normalized scores. As the observed difference increases, the calculated score decreases, indicating a lesser probability of encountering large charge differences during molecular alignment. Interestingly, the slope of the graph is significantly steeper within the range of 0–1.2, reflecting a more rapid decline in score as the observed difference increases within this interval. Beyond 1.2, up to 3, the slope becomes less steep, indicating a more gradual decrease in score with increasing observed difference. This inflection at 1.2 could suggest that smaller charge differences (less than 1.2) are more common in the dataset, while larger differences (greater than 1.2) occur less frequently.

Table 1 presents all outcomes for every possible parameter combination derived from the all-versus-all scoring matrix for both Levenshtein similarity (subfigure A) and the difference between Levenshtein and Exact similarities (subfigure B) in heat maps. A standout observation from these subfigures is the high similarity average in Levenshtein similarity, which surpassed 0.94, and a corresponding low difference between the two string similarities (0.05894) for certain parameter combinations, underlining a marked degree of similarity amongst the aligned sequences.

**Table 1:**
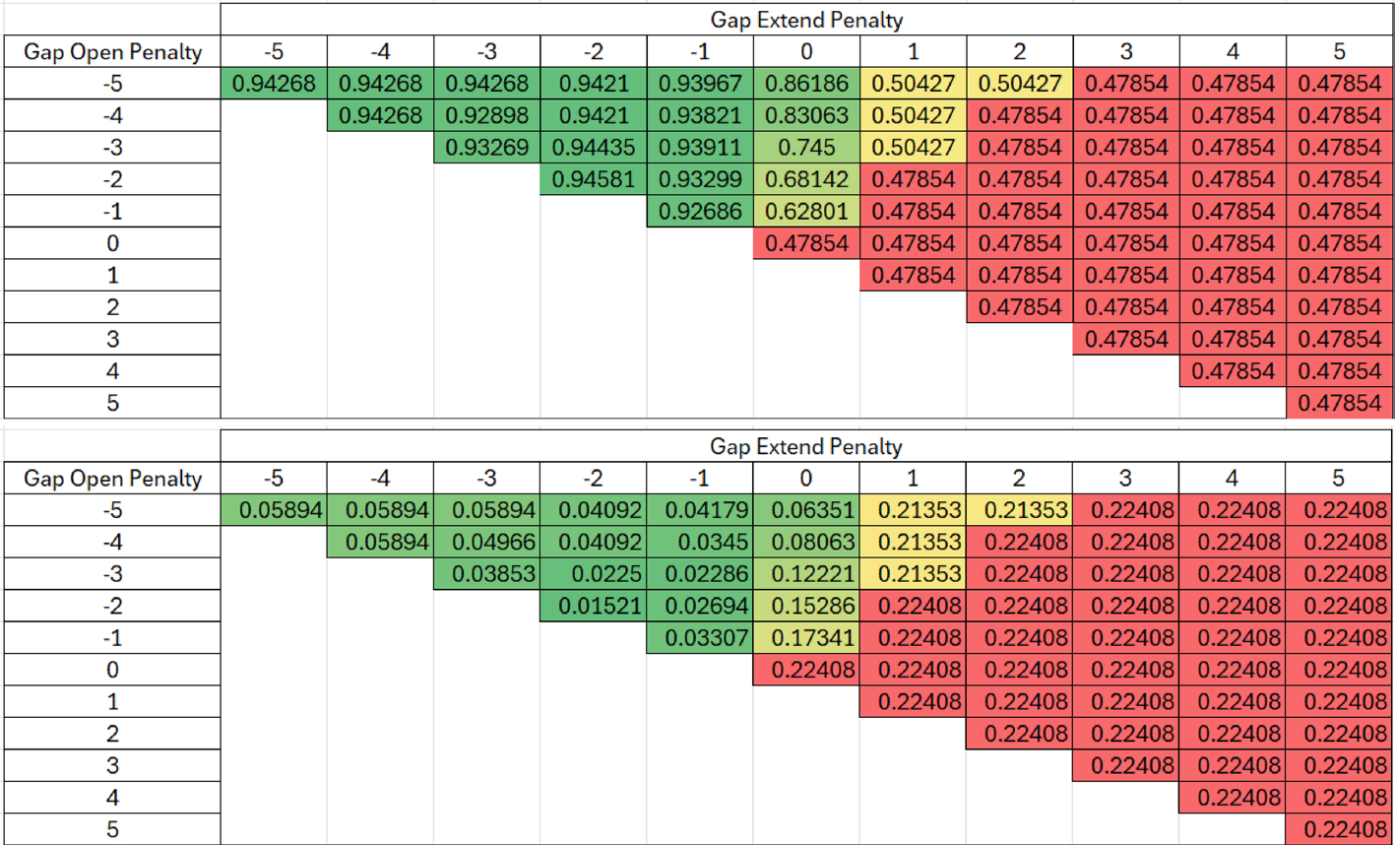
Heat Map of all Parameter Combinations in the All vs All Scoring Matrix for the Krebs cycle Truth Dataset with Levenshtein (Top) and the Difference between Levenshtein and Exact (Bottom). The All vs All scoring alignment algorithm was validated using the truth dataset and determined using Gap open and gap extension penalties ranging from − 5 to + 5. The accuracy of the alignment was measured using Levenshtein similarity in Table 1a. The difference between Levenshtein and exact similarities are shown in Table 1b to evaluate any systematic change in exact exact similarity

### Paired scoring and alignment

**Fig. 5:**
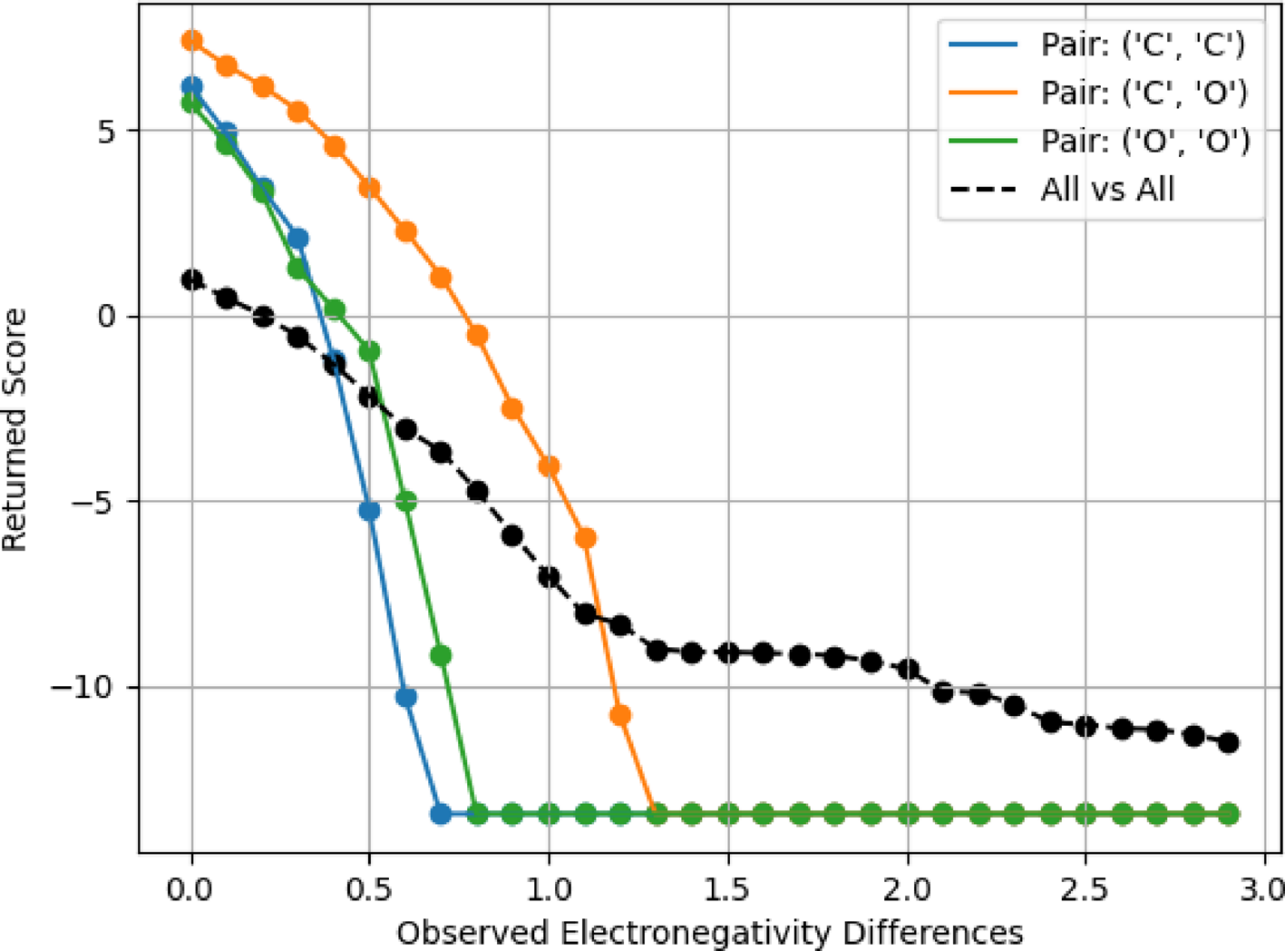
Common Pairs in the Paired Scoring Matrix and All vs. All Scoring Matrix. Log2 probability of observed Gasteiger charge differences or greater in 0.1 increments of each atom pair in the database

Figure 5 presents a graphical comparison between the scoring matrices for the most frequently observed atom pairs (‘C’, ‘C’), (‘C’, ‘O’), and (‘O’, ‘O’) and the all-vs-all scoring matrix derived in the earlier section.

The plots for the atom pairs exhibit an inverse quadratic shape initially, suggesting a nonlinear correlation between charge differences and their corresponding scores for small charge differences. However, these plots transition into a line once the scoring matrix reaches its largest penalty, which appears to be at a score of around − 15. This shift reflects the enforcement of the largest penalty for extreme charge differences. In contrast, the all-vs-all scoring matrix plot maintains a different profile. It displays an initial steep linear decline that flattens out for larger charge differences, with the maximum penalty positioned approximately at − 12.5. This divergence from the atom pair-specific plots underscores the distinctive characteristics of the all-vs-all scoring matrix, reflecting a broader spectrum of atom types and charge differences.

Table 2 presents all outcomes for every possible parameter combination derived from the paired scoring matrix for both Levenshtein similarity (subfigure A) and the difference between Levenshtein and Exact similarities (subfigure B) in heat maps. Both subfigures share high Levenstein similarities and correspondingly low differences between Levenstein and Exact similarities for certain parameter combinations, once again demonstrating similar responses in similarity methods.

**Table 2:**
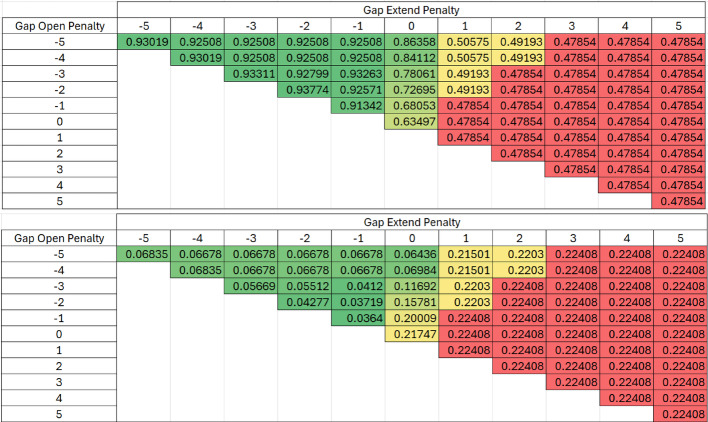
Heat Map of all Parameter Combinations in the Paired Scoring Matrix for the Krebs cycle Truth Dataset with Levenshtein (Top) and the Difference between Levenshtein and Exact (Bottom). The Paired scoring alignment algorithm was validated using the truth dataset and determined using Gap open and gap extension penalties ranging from − 5 to + 5. The accuracy of the alignment was measured using Levenshtein similarity in Table 2a. The difference between Levenshtein and exact similarities are shown in Table 2b to evaluate any systematic change in exact similarity

### Glycolysis valdiation

The same method used for validation with the Krebs cycle was also applied to glycolysis. Tables 3 and 4 presents all outcomes for every possible parameter combination derived from the all-versus-all and paired scoring matrix, respectively, for both Levenshtein similarity (subfigure A) and the difference between Levenshtein and Exact similarities (subfigure B) in heat maps. The best Levenshtein averages are 0.14 and 0.16 points lower for the all vs all and paired scoring matrices, respectively, compared to their Krebs cycle validation counterparts. The lowest differences between Levenshtein and exact similarities are 0.09 points higher for both the all vs all and paired scoring matrices. These discrepancies show slightly different performance on the two pathways.

**Table 3:**
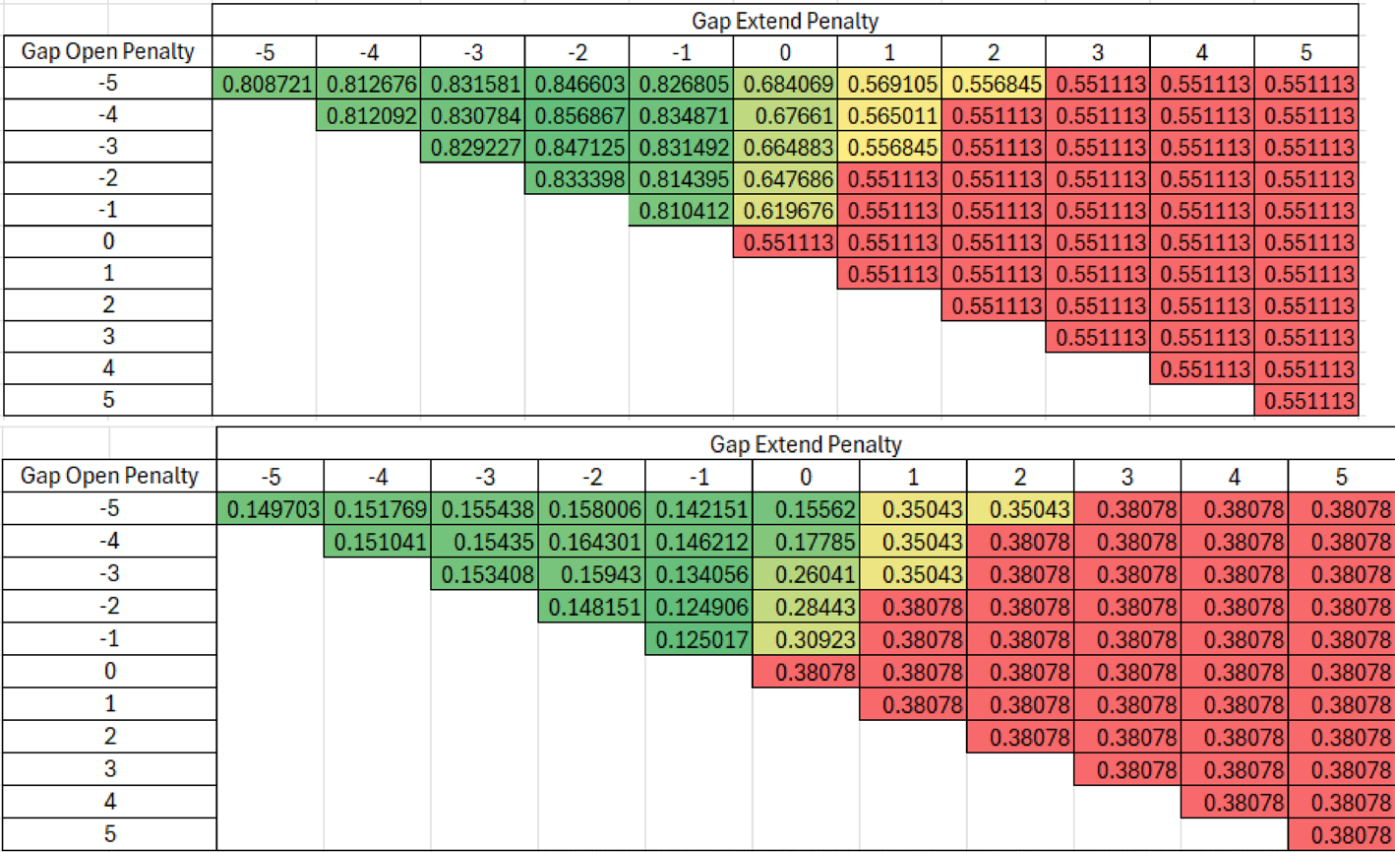
Heat Map of all Parameter Combinations in the All vs All Scoring Matrix for the Glycolysis Truth Dataset with Levenshtein (Top) and the Difference between Levenshtein and Exact (Bottom).The All vs All scoring alignment algorithm was validated using the Glycolysis truth dataset and determined using Gap open and gap extension penalties ranging from − 5 to + 5. The accuracy of the alignment was measured using Levenshtein similarity in Table 3a. The difference between Levenshtein and exact similarities are shown in Table 3b to evaluate any systematic change in exact similarity

**Table 4:**
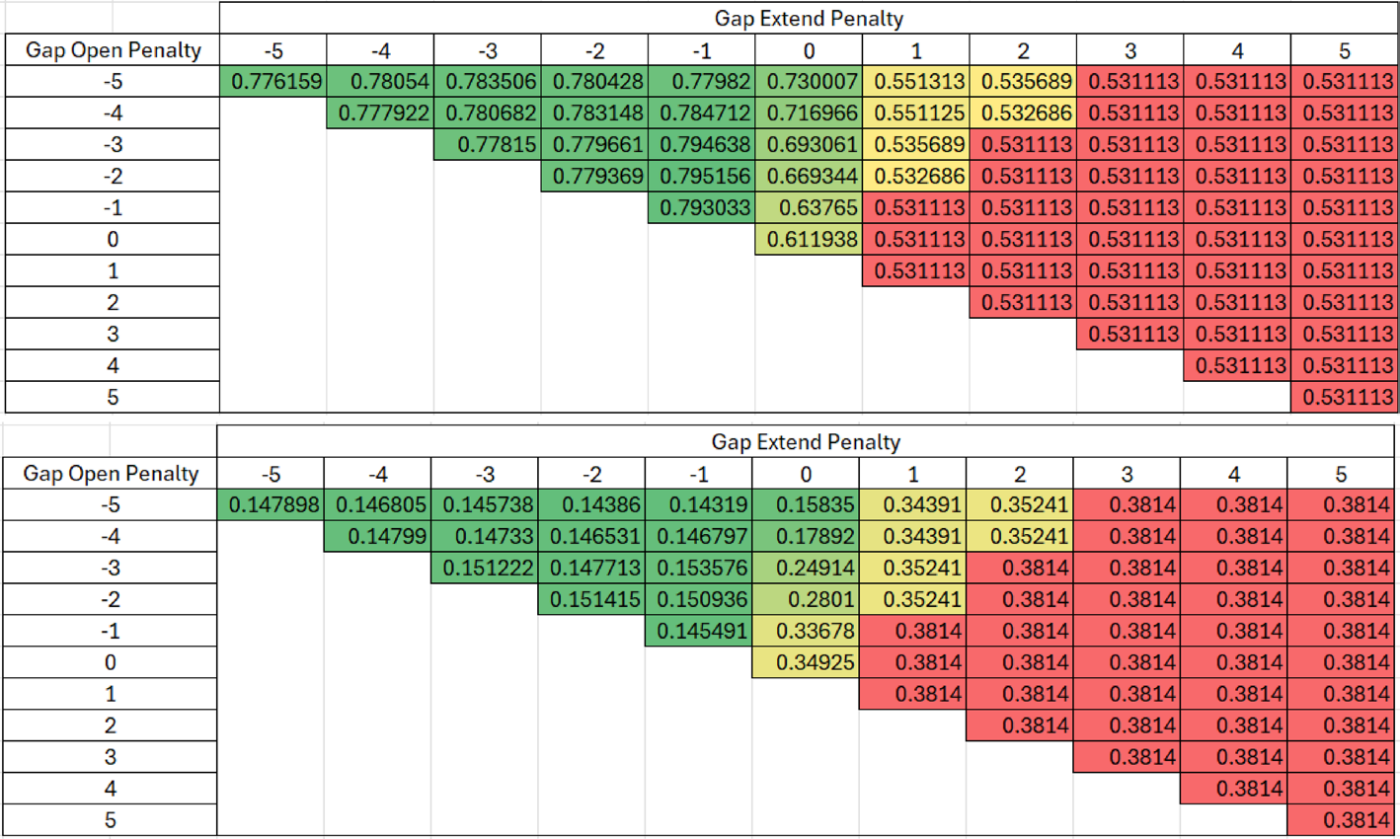
Heat Map of all Parameter Combinations in the Paired Scoring Matrix for the Glycolysis Truth Dataset with Levenshtein (Top) and the Difference between Levenshtein and Exact (Bottom). The Paired scoring alignment algorithm was validated using the Glycolysis truth dataset and determined using Gap open and gap extension penalties ranging from − 5 to + 5. The accuracy of the alignment was measured using Levenshtein similarity in Table 4a. The difference between Levenshtein and exact similarities are shown in Table 4b to evaluate any systematic change in exact similarity

### Application

Two prominent models, the retrograde and patchwork evolutionary models, offer distinct perspectives on the evolution of metabolic pathways [29, 31, 32]. The retrograde model posits that metabolic pathways adapted to the scarcity of specific substrates by developing new enzymes for more abundant, upstream compounds [29, 31]. 

In contrast, the patchwork model suggests that early enzymes with broad substrate specificity gradually specialized through processes like gene duplication and sequence divergence [29, 32]. For some chemical processes, including those that generate ATP, linear pathways can become cyclical ones under some selective constraints, but also with chemical constraints as well.

In the evolution from linear to cyclical pathways, a key characteristic is that molecular transformations do not alter the molecule so drastically that it cannot revert to its original structure; such an extreme change would result in the pathway remaining linear. In a cyclical molecular pathway, how significant can the chemical transformations of any starting molecule be at each step before it returns to its original position? Molecules become increasingly dissimilar from their initial structure up to a certain point in the cycle, after which they begin to revert. Because the algorithm developed quantifies these transformations to generate a global alignment of small molecules, it allows for the tracking of molecular changes, pinpointing key transformation steps, and identifying when molecules start resembling their original state. Furthermore, it facilitates comparative analysis across different pathways, potentially uncovering patterns in the evolutionary shift from linear to cyclical pathways.

The Pentose Phosphate Pathway (PPP) is a vital metabolic route that functions parallel to glycolysis. It chiefly assists in the generation of ribose-5-phosphate, which is vital for nucleotide synthesis, and in the production of NADPH, an indispensable cofactor for reductive biosynthesis reactions within cells [28]. Utilizing both the all vs all and paired scoring algorithms on this pathway, as retrieved from the KEGG database [33], provides insights into molecular transformations throughout the PPP.

**Fig. 6:**
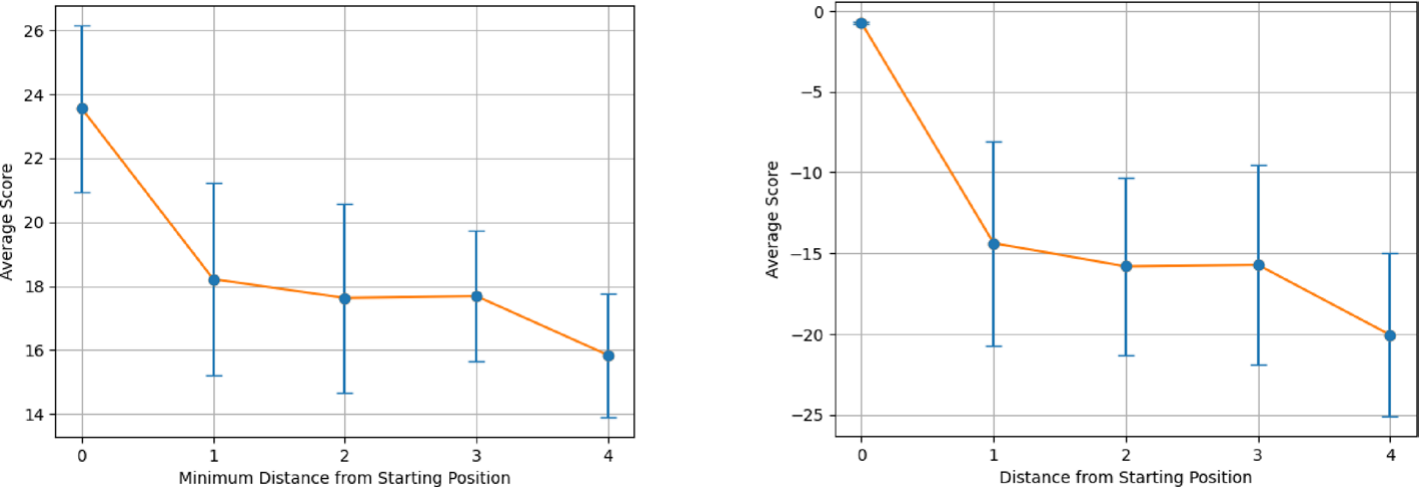
Alignment of the PPP using all vs all scoring matrix. Using the best parameters (0 for both gap open and gap extension determined in the validation step), each step of the cyclical pathway was aligned with the subsequent steps so that the entire cycle is aligned for each step. The y axis shows the average score for each distance from the starting position (x axis). Standard Deviations [2.60, 3.01, 2.95, 2.03, 1.94]

**Fig. 7:**
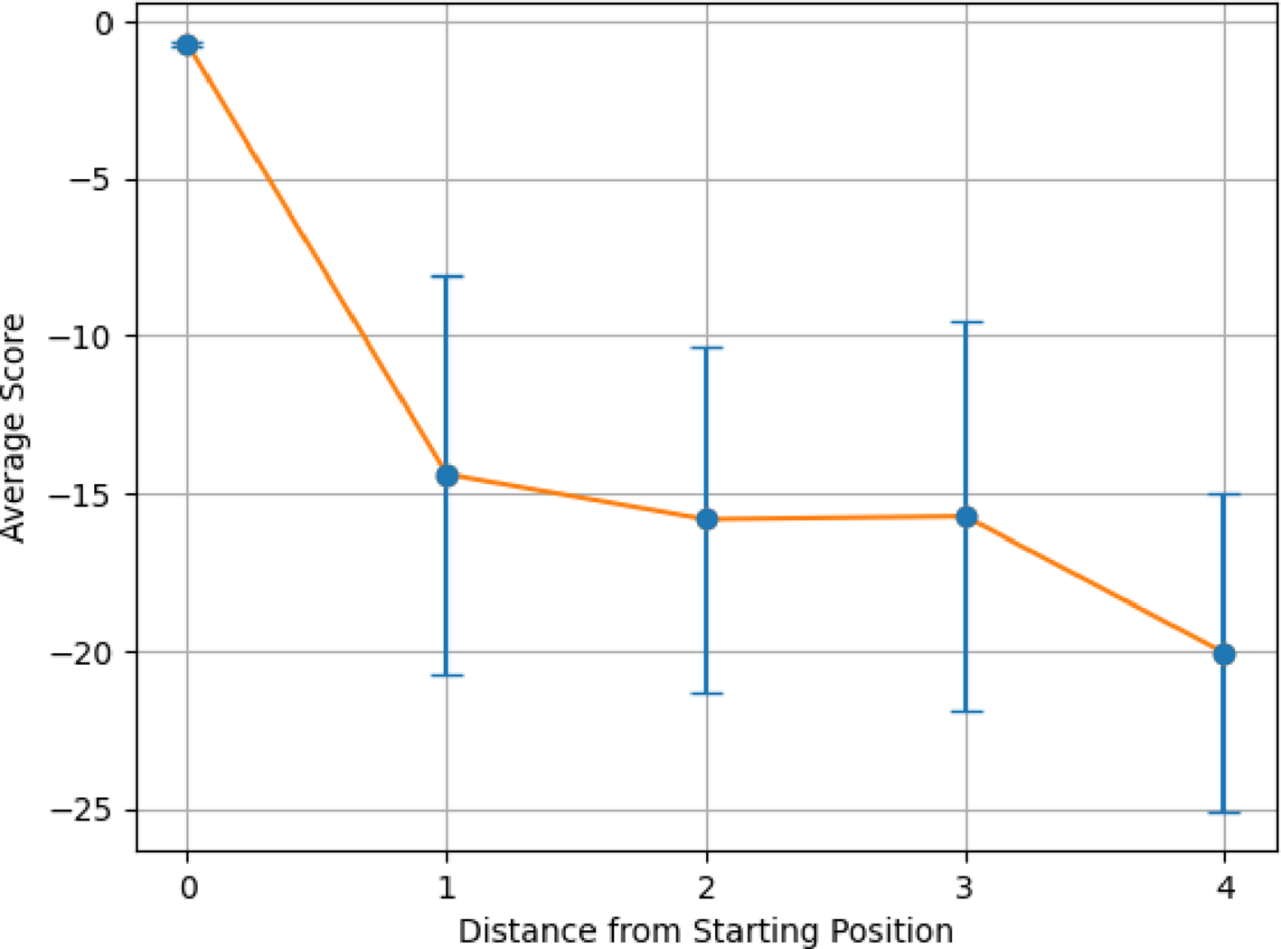
Alignment of the PPP using paired scoring matrix. Using the best parameters (− 2 for both gap open and gap extension determined in the validation step), each step of the cyclical pathway was aligned with the subsequent steps so that the entire cycle is aligned for each step. The y axis shows the average score for each distance from the starting position (x axis). Standard Deviations: [0.078, 6.33, 5.52, 6.21, 5.04]

**Fig. 8:**
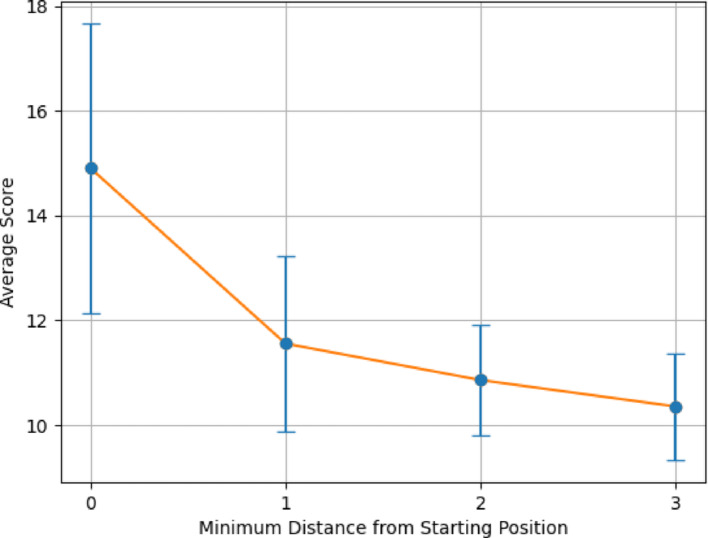
Alignment of the Krebs cycle using all vs all scoring matrix. Using the best parameters (0 for both gap open and gap extension determined in the validation step), each step of the cyclical pathway was aligned with the subsequent steps so that the entire cycle is aligned for each step. The y axis shows the average score for each distance from the starting position (x axis). Standard Deviations: [2.76, 1.68, 1.06, 1.02]

**Fig. 9:**
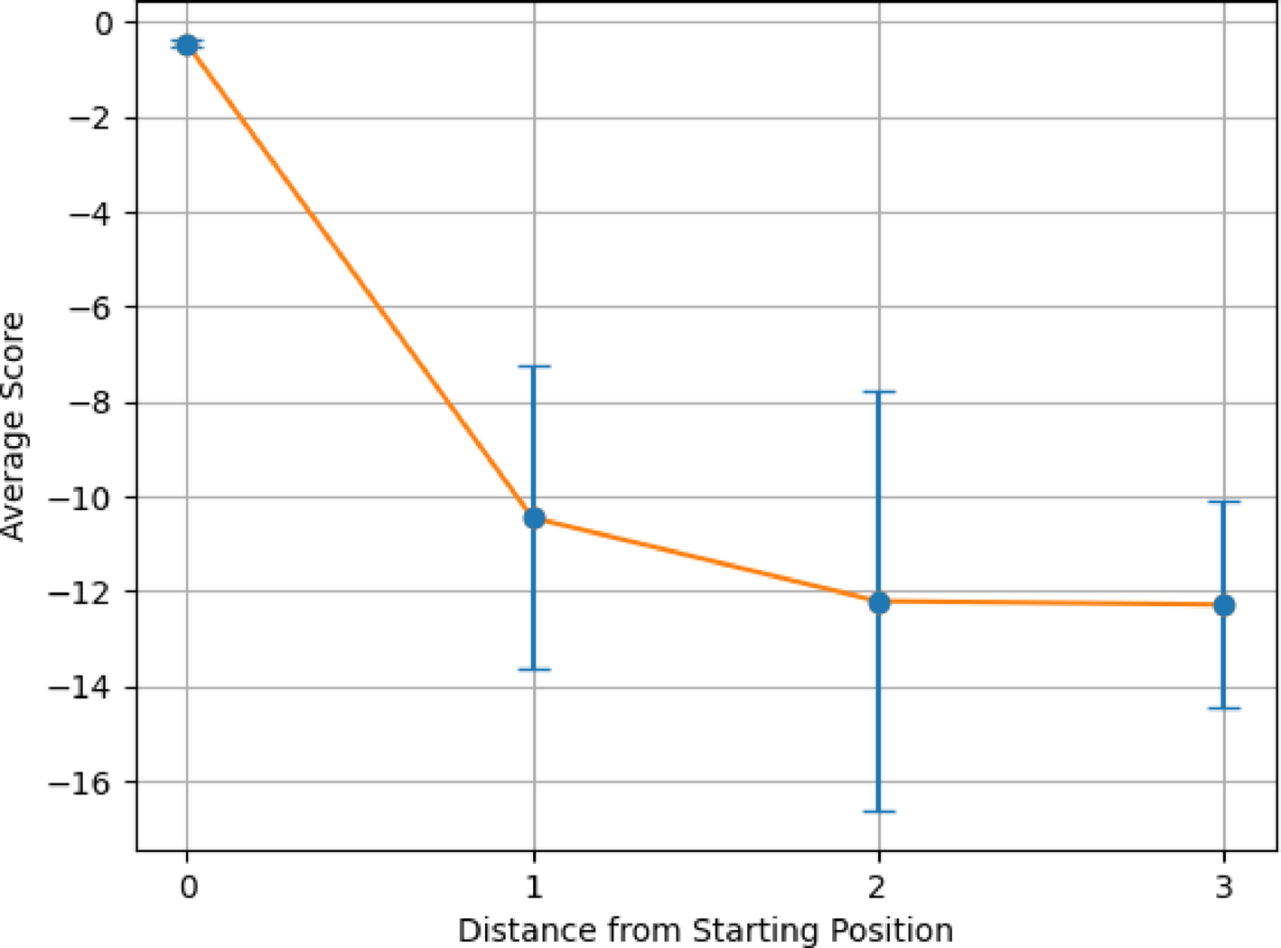
Alignment of the Krebs cycle using paired scoring matrix. Using the best parameters (− 2 for both gap open and gap extension determined in the validation step), each step of the cyclical pathway was aligned with the subsequent steps so that the entire cycle is aligned for each step. The y axis shows the average score for each distance from the starting position (x axis). Standard Deviations: [0.085, 3.19, 4.42, 2.19]

Figures 6 and 7 demonstrates the average alignment score for each minimum distance in the cycle for the best parameters found in the validation for each step in the pathway, for both All vs All and Paired Scoring methods, respectively. Both figures follow the expectations of a cyclical cycle similarity: descending in similarity with an initial score of 23.5 for all vs all and − 0.7 for paired, reaching a point of most dissimilarity (Distance 4 in Figs. 6 and 7) with a score of 15.8 for all vs all and − 20.1 for paired, and approaching greater similarity as the transformations return to its starting position (Distance 0 in Figs. 6 and 7). For both scoring methods, the most dissimilar positions appear most distant from a given position. For comparison, the Krebs cycle was analyzed using the same parameters (Figs. 8 and 9), and demonstrates the expectation described above and that is seen in the PPP with a starting score of 14.9 for all vs all and − 0.5 for paired, and final score of 10.3 for all vs all and -13.8 for paired. The Krebs cycle alignments also show that the most dissimilar part of the cycle is also the most distant position (Distance 3 in Figs. 8 and 9). The error bars show high variance for both figures, likely due to the different sizes of the molecules which have large impacts on the alignment scores. It is possible for large pathways that molecules will asymptote out at a high level of distance.

**Fig. 10:**
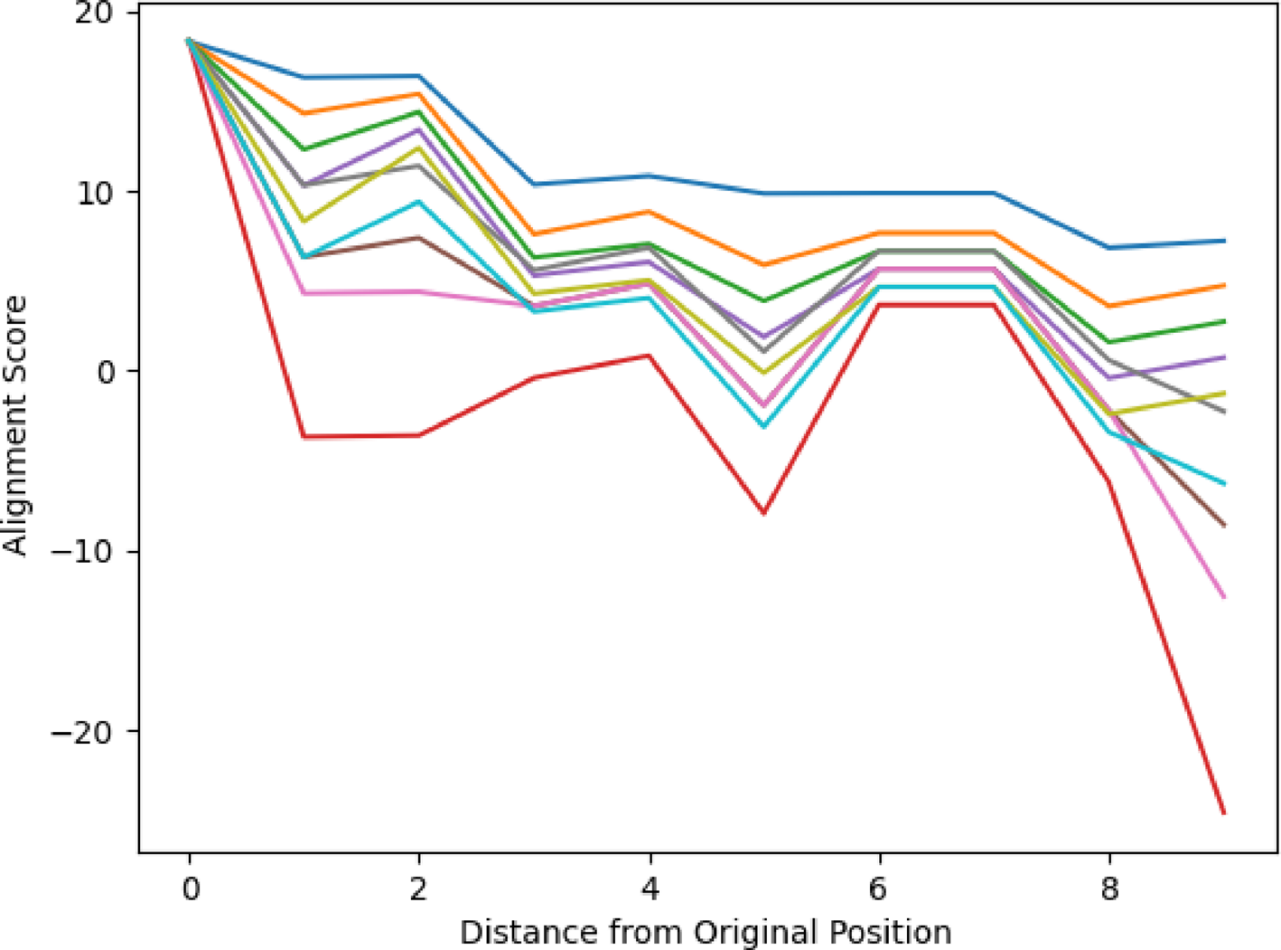
Alignment of Glycolysis Pathway using All vs All Scoring. Using the top 10 parameters, the linear pathway glycolysis was aligned using the All vs All scoring algorithm, demonstrating the algorithm’s ability to show decreasing alignment scores expected in linear pathways

**Fig. 11:**
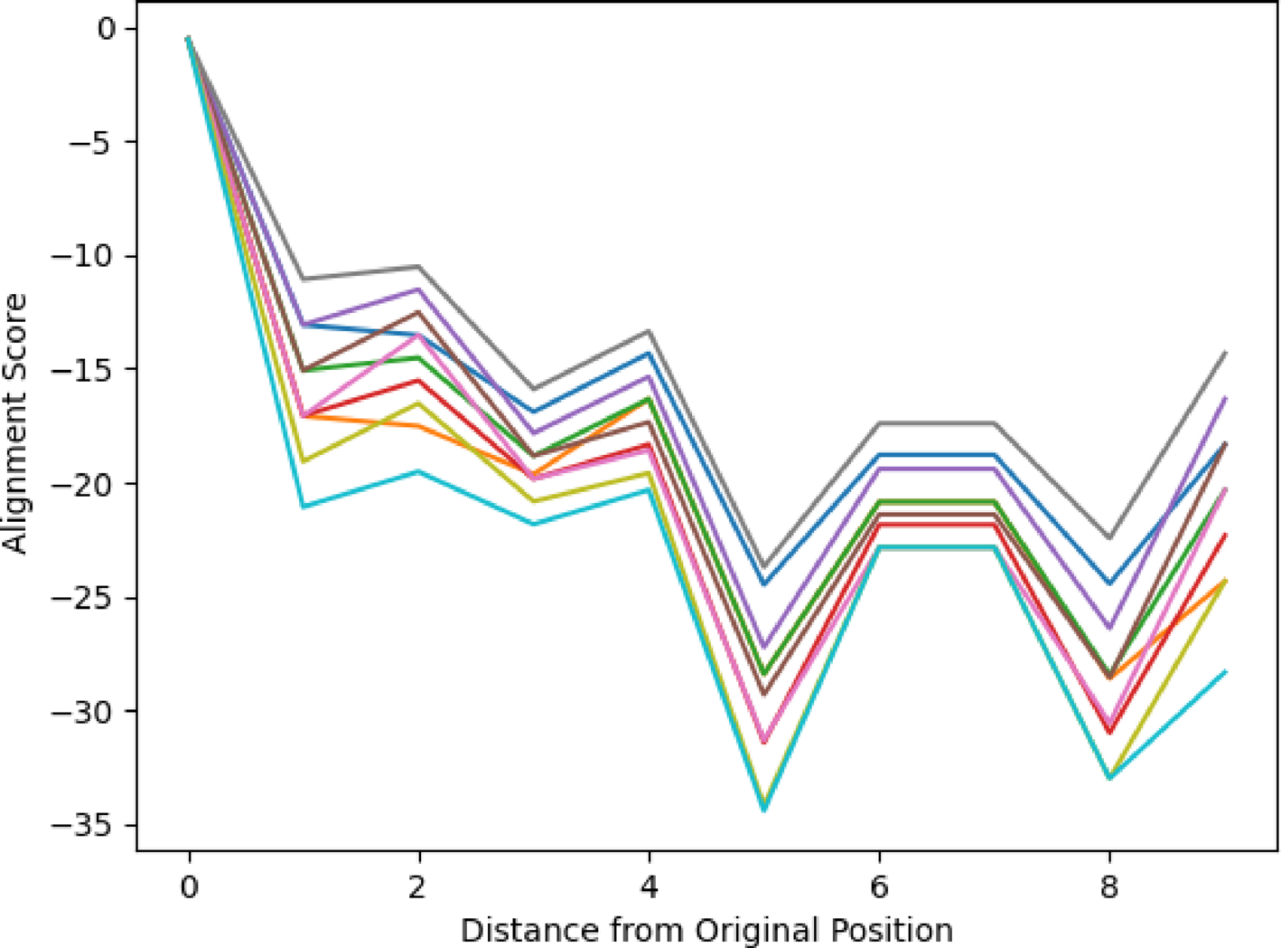
Alignment of Glycolysis Pathway using Paired Scoring. Using the top 10 parameters, the linear pathway glycolysis was aligned using the Paired scoring algorithm, demonstrating the algorithm’s ability to show decreasing alignment scores expected in linear pathways

Figures 10 and 11 display the alignment scores for the Glycolysis pathway using All vs All and Paired Scoring methods, respectively. Consistent with the expected behavior of a linear pathway, the figures illustrate an increase in dissimilarity as the distance from the starting molecule grows. The nonlinearity of the changes reflects the underlying chemistry of the transformations. This trend in the alignment scores reaffirms the algorithm’s validity, particularly for the All vs. All scoring in Fig. 10, complementing the patterns observed in Figs. 6, 7, 8 and 9 for cyclical pathways, where dissimilarity peaks most distant from the starting point in the cyclical pathway (Distance of 4 in PPP, and Distance 3 in Krebs Cycle) and then decreases returning to the starting point in the pathway (Distance 0). Since cyclical pathways don’t have explicit start and end points, the pathways were aligned and plotted in a way to be the average of each distance in the pathway, where the first index of scores is the average score of each molecule with itself. The second index is the average of scores of each molecule with subsequent and previous molecules in the pathway. This is done until each distance has been measured. The graph only shows half of the number of molecules in a pathway since the minimum distance is considered in both directions. Collectively, these results validate the algorithm’s efficacy in capturing the anticipated divergence patterns in both linear and cyclical metabolic pathways. It should be noted that the pentose phosphate pathway has both a linear and a cyclical form and is being treated as cyclical here.

**Fig. 12:**
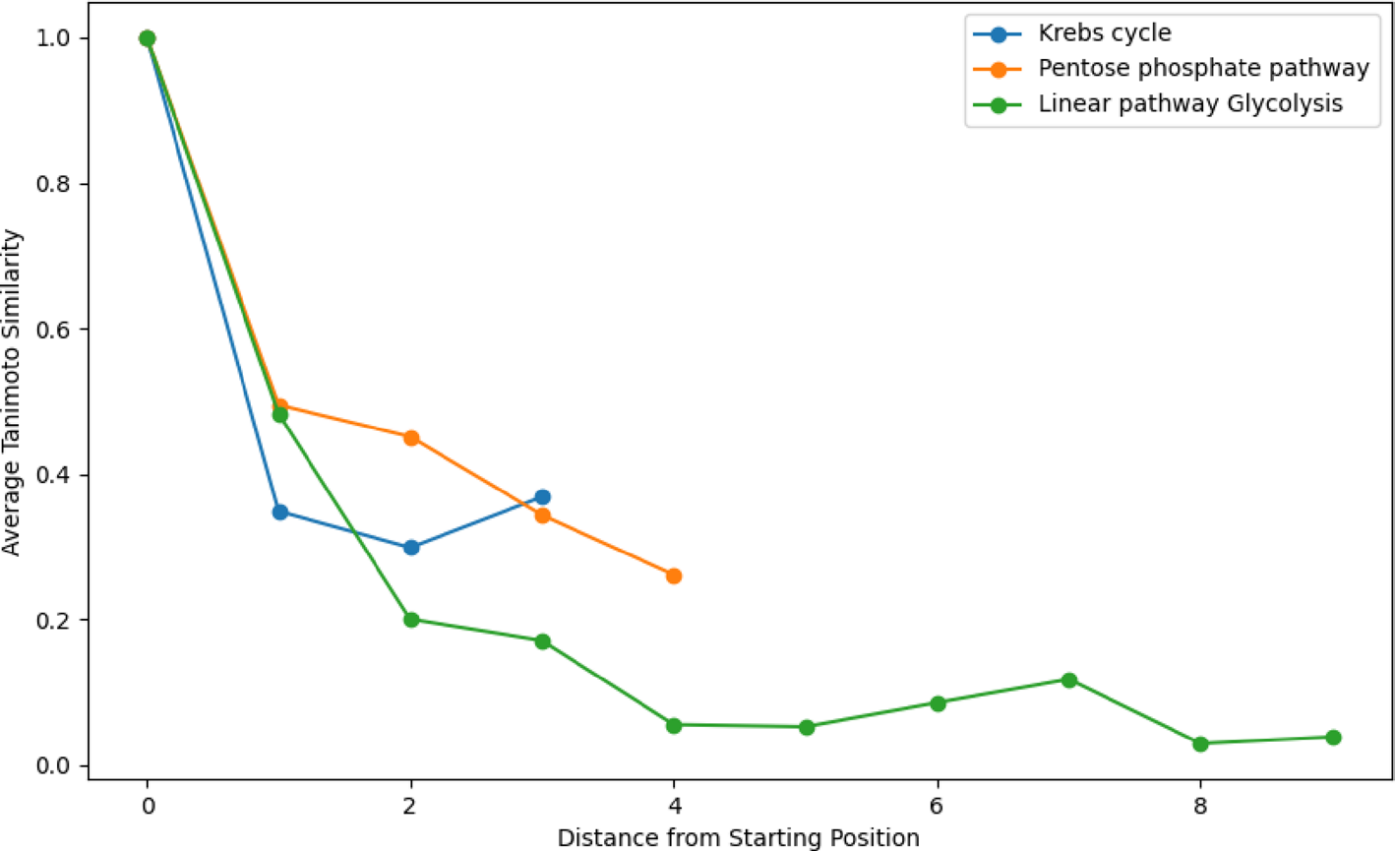
Levenshtein vs Exact Averages in Paired Scoring plots the Tanimoto coefficient score for molecules in the Krebs cycle, Pentose phosphate pathway, and Glycolysis as a means of comparison with the novel algorithm

Figure 12 illustrates the average Tanimoto similarity scores for the Krebs cycle and the Pentose phosphate pathway as a function of the minimum distance from the starting position. The linear pathway Glycolysis was also calculated using the Tanimoto similarity of each molecule with the starting molecule. The measure does not consider the dynamic nature of metabolic networks or the influence of environmental factors on pathway evolution. Tanimoto similarity might not capture the full complexity of biochemical transformations and regulatory mechanisms within these pathways. However, this method can still provide valuable insights. By comparing the average Tanimoto similarity scores, we can identify trends and potential evolutionary relationships between pathways. While not definitive, these comparisons can highlight similarities that warrant further investigation, aiding in our understanding of the evolutionary context of metabolic pathways. Figure 12 shows our original assumption that similarity decreases then increases from a given starting position to its most distant with cyclical pathways, particularly the Krebs Cycle, and a general decreasing with linear pathways. The results from the pentose phosphate pathway are not consistent with those from the Krebs Cycle. This is consistent with results from the alignment method.

## Discussion

The original Needleman-Wunsch algorithm, being a well-established method in the field of bioinformatics, has been made widely available in various scientific programming libraries [24]. Its general simplicity, combined with the documentation available, ensured that its integration into the pipeline was significantly expedited. The development of the custom SMILES alignment algorithm, seen as an expansion on the Needleman-Wunsch, was facilitated efficiently due to its foundational reliance on an existing approach [24].

In the alignment process for molecules represented as SMILES, the decision to strip non-atomic characters was driven by the need to distill the essence of the molecule to its electronegativity blueprint. SMILES notations are rich in detail, encompassing both atomic and non-atomic characters. While this offers a comprehensive representation, it introduces the challenge of aligning non-characterizable entities, which would introduce unnecessary noise during the alignment process. By eliminating these characters, the focus shifts entirely to the alignment of the underlying partial charge patterns intrinsic to each atom. It’s pertinent to note that while explicit characters indicating certain molecular features are absent post-stripping, the retained partial charge is not an isolated characteristic; it’s deeply influenced by both the atom type, bond type, and its spatial orientation within the molecule. Thus, the alignment process, by focusing on this partial charge blueprint, effectively captures the core nature and orientation of atoms within molecules, ensuring a more refined and accurate alignment devoid of the potential distractions introduced by non-atomic characters.

The research and subsequent results on the alignment algorithms notably bridge a gap in the fields of chemoinformatics and bioinformatics. While both domains have seen rapid advancements, the specific challenge of molecular alignment, especially as it pertains to intricate biochemical pathways like the Krebs cycle, has largely remained a complex puzzle. This study’s achievements offer a tailored solution, catering not only to the general alignment needs but also to the subtleties and nuances of biochemical transformations. By building upon and refining established alignment techniques, this tool provides an optimized approach, meticulously adapted to chemical structural data.

There are limitations to the algorithm that need to be considered. For instance, the algorithm is extremely sensitive to the format of the SMILES strings as without canonization, the molecule may yield inconsistent and unreliable alignment scores. Therefore, proper data processing is necessary. Consideration of cofactors is also a limitation that can be relaxed in future work. As the method is developed further, more informative chemical metrics beyond those employed here may produce improved scoring matrices.

The algorithm has unveiled that within cyclical metabolic pathways, the point of greatest dissimilarity from the starting molecule typically occurs halfway through the cycle. This discovery suggests a limit of molecular alteration beyond which a linear pathway may not naturally evolve into a cyclical one that potentially creates barriers to cyclize for longer pathways with more transformations. Moreover, the analysis indicates that in linear pathways, similarity diminishes as the distance from the starting molecule increases, implying an inverse relationship between similarity and pathway progression. These findings could help in predicting the evolutionary potential of metabolic pathways, providing a metric to gauge the likelihood of a pathway remaining linear or becoming cyclical based on its molecular transformation scores.

For instance, how might a particular molecule evolve within a metabolic pathway? How can we predict structural transformations in newly discovered or lesser-known pathways? What are the likely structural consequences of introducing a novel compound into a system? These are all questions that can be addressed with the collection of appropriate data and the application of the approach presented here. The algorithm has the potential to systematically analyze molecular libraries, highlighting evolutionary trends and pinpointing the evolutionary shifts in metabolism. Using large libraries, it can be determined what the threshold for similarity is for cyclical pathways. Furthermore, its predictive power could be leveraged to infer the metabolic roles of molecules that are not yet understood, by evaluating their similarities within the extensive network of metabolic reactions. Additionally, the approach could be extended to enable molecular search in large databases. Such capabilities could significantly enhance our comprehension of metabolic evolution and the intricate design of biological systems.

## Conclusion

The presented algorithm is a freely available open-source tool that facilitates precise molecular alignment using the Simplified Molecular Input Line Entry System (SMILES) format. By leveraging dynamic programming and incorporating partial charge measurements, this tool effectively aligns small organic molecules and quantifies molecular transformations in metabolic pathways. Its application to benchmark datasets, such as the Krebs cycle, glycolysis, and the Pentose Phosphate Pathway, demonstrates its robustness and utility in biochemical and molecular research.

## Availability and requirements

- Project name: SMILES Alignment
- Project home page: https://github.com/24atang/SMILES-Alignment.git
- Operating systems: POSIX-like operating systems (OS X, Linux)
- Programming language: Python
- Other requirements: RDKit, PubChem
- License: None
- Any restrictions to use by non-academic: None

## Data Availability

The datasets analysed during the current study are available in the REACTOME database (https://reactome.org/PathwayBrowser/#/R-HSA-1430728) and from PubChem (https://www.ncbi.nlm.nih.gov/pccompound). The datasets generated during the current study are available in the SMILES-Alignment repository https://github.com/24atang/SMILES-Alignment.git.

## Abbreviations

SMILES: Simplified molecular input line entry system
HTS: High throughput screening
AAP: Atom-atom-path
MCS: Maximum common subgraph
NaN: Not a number
PPP: Pentose phosphate pathway
MOS: Maximum overlapping set
POA: Partial order alignment
